# Local governance of sport: a national survey on how public opinion evaluates municipal sports development and priorities

**DOI:** 10.3389/fspor.2026.1751428

**Published:** 2026-02-19

**Authors:** André Dionísio Sesinando, Jordi Seguí-Urbaneja, Mário Coelho Teixeira

**Affiliations:** 1 University of Évora, Évora, Portugal; 2 Center for Advanced Studies in Management and Economics - CEFAGE-UÉ, University of Évora, Évora, Portugal; 3 National Institute of Physical Education of Catalonia - INEFC, University of Lleida, Lleida, Spain

**Keywords:** sport management, developing sports, sport governance, sport policies, local authorities, public spending, perceived satisfaction, public opinion

## Abstract

Public policies in sport and good governance adopted by local authorities aim to promote healthier habits and widespread participation in sport. However, little is known about the use of policy instruments by Portuguese municipalities to assess its implementation, evaluation and monitoring. At the same time, there is insufficient data available to evaluate political action and local sports development, making it difficult to assess this progress. Therefore, this research was designed to measure satisfaction with the current level of development in municipalities according to different sports development indicators. We followed an exploratory methodology using quantitative-descriptive and inferential analysis. Data was collected through a questionnaire between November 2024 and January 2025 with 937 responses obtained after validation. Results show a level of agreement between genders regarding satisfaction with the level of sports development, sports services and facilities, while transparency does not show encouraging results due to high level of non-formed opinion. There is also a high level of agreement on policy priorities and the influence of political orientation on public investment in sports. Municipalities play a key role in promoting local sports and modernizing cities and sport facilities, being essential to keep studying their action in different perspectives to measure good governance in sports development and political transparency perception.

## 1 Introduction

Research in sport management has been progressively advancing, receiving wide recognition and merit for deepening knowledge about the functioning and structure of sports organizations (1, 2). Whether in the public or private sector, the level of intervention has expanded and is now more complex, multidisciplinary and multilevel, making it imperative to continue in-depth study to better understand and respond to a wide range of issues in modern sports (3). However, existing knowledge about municipal intervention in local sports development, as well as their role as the reference in the promotion of sport, provision of services and public investment in sports facilities is still limited from the point of view of citizens' opinions, particularly in the field of sport management (4).

As a contribution to greater understanding of this important issue in Europe, while also incorporating an approach based on good governance and transparency in sport, an opportunity was identified to design a study to analyze citizens' opinions as an indicator of local sports development. The main objective of this research was to assess citizens' opinions and the level of agreement about different aspects within the scope of study about local development and political action, in order to explore the current state of satisfaction with local sports development in municipalities.

## 2 Literature review

### 2.1 Local sports development: a perspective from sport management

Sport management, as an area of knowledge and unique tool in the study of sports organizations and the development of sport from different perspectives, enables us to better understand the role of municipalities in providing access to and promoting sport in various social contexts (3, 5). Although existing knowledge reveals a lack of multidisciplinary studies produced in the last decade (4), many authors who have followed this topic are consistent in stating that local authorities are effectively the driving force behind local, regional, and national sports development in Europe (6, 7), especially in smaller countries with less economic power, such as Portugal (8).

The model of sports development in Europe, as established in the 2007 Treaty of Lisbon and recently reinforced by the European Parliament through the adoption of resolution P10_TA(2025)0212 entitled “*Role of EU policies in shaping the European model of sport*”, formally recognizes sport as a fundamental pillar of modern society and a distinguishing factor due to its social and educational role (9). Ideologically, this model is based on solidarity, sporting merit, and decentralized organization (6), while also valuing the role of volunteering and sport as an educational tool in the adoption of healthier habits, as well as more active lifestyles throughout citizens' lives (3, 10).

In comparative terms, the promotion of sport is based on two distinct models: the American model, which focuses on boosting the business of sport at all levels, as opposed to the European model, which focuses on the public promotion of sport as an instrument for the progress of civilization. This means that it is up to national governments to adopt policies that respect the European vision of sports development (7), acting not only as legislators, but above all as the main agents of promotion and investment in creating conditions for access to sports for all citizens, without exception (11, 12).

In this matter, the public sector should be the driving force behind the development of sport and sporting activities (5), with the central government delegating to its organic structures and substructures a range of comprehensive, well-defined and strategically planned responsibilities and powers to better serve the population (13), in order to ensure and adjust public investment in line with the needs and opportunities of each specific region (14). In this context, municipalities are objectively the main reference point due to their proximity to citizens (15), enabling them to respond appropriately to existing concerns and needs, as well as working towards higher levels of satisfaction among citizens regarding the level of development in the municipality where they live (4, 16).

When it comes to Portugal, local government intervention in the field of sport is multidisciplinary and multilevel, covering responsibilities in defining and planning public policies, as well as implementing and evaluating them (4, 6). As such, their daily activities include: a) the construction and maintenance of public sports facilities and venues; b) the planning and implementation of annual, monthly, and/or one-off recreational and sporting activities; c) the implementation and support for the organization of sporting events of different types of their own initiative or in association with private entities; d) operating and offering municipal sports services in municipal sports facilities (swimming pools, athletics centers, sports halls, among others); e) providing logistical and financial support to sport and recreational associations; f) supporting and offering sports activities in public schools, especially in preschool and primary education; g) auditing sports activities promoted by the private sector; h) promoting the municipality worldwide to attract international competitions and events; i) promoting the municipality in applications for awards; and j) among others (13).

We can therefore recognize that the range of intervention of Portuguese municipalities is now much broader and complex than initially envisaged in the process of decentralization of powers and local autonomy (8, 17), not only due to Portugal's democratic maturation process, but especially due to the need to adjust and align with European standards and common practices in terms of how the state is organized (18). This process has provided the conditions for better governance of public resources and enabled the State to make efficient and effective decisions regarding the adoption of public policies (18), but also more difficult to balance citizens' expectations and good financial management of municipalities (19), especially when it comes to public sports policies (3, 20).

Despite the increased accountability and scrutiny of municipal activities in Portugal, and the existence of instruments validated by the international scientific society for evaluating public policies (21, 22), we find a consensus among different authors regarding the continued absence and lack of public disclosure of results obtained with certain public policies (23), including sport policies (24). This lack of information and the failure to present results that are absolutely relevant for studying and evaluating local sports development, not only contributes to increasing the perception of transparency and opacity in the choices made by Portuguese municipalities (25), but above all validates the widespread perception in society that many policy measures are designed and implemented without a proper methodology and evaluation control, making it difficult to assess the level of local sports development in the various regions of Portugal with any degree of quality and accuracy (14).

However, we live in a society that is more vigilant and aware of political power and its actions at various levels, especially when it comes to local authorities (26), where proximity is greater and the impact on perceptions about cities development is more visible to the population (27). This awareness is now having an increasing impact on political scrutiny over the actions taken, as well as on the decision-making process (28), exerting greater pressure not only on the activities of the municipalities themselves, but above all on constant communication about local political decisions.

This level of engagement suggests that citizens are increasingly concerned about the role played by elected politicians in improving and developing cities, as well as what they offer the population (16). Residents' satisfaction is regularly used as a tool to measure and evaluate their actions and priorities (29), making public opinion increasingly important as the main indicator of (dis)satisfaction with local and national political decisions (30, 31), while also exerting considerable power over the public image of political elites and political parties (30).

Nevertheless, in the absence of use (whether deliberate or not) of assessment tools capable of measuring the effectiveness and efficiency of certain public policies in Portugal, as well as the level of local development, especially in relation to sport, it is recognized that public opinion has been used as an indicator for assessing satisfaction and well-being. The reason for this is that local elected representatives and political parties constantly need to be aware of citizens' sensitivities and opinions regarding whether they agree or disagree with the decisions taken during the governing period (31).

In the absence of available data and satisfactory results to understand this phenomenon, polling has been repeatedly used as a means of assessing levels of agreement and satisfaction in different contexts (27), including in the field of sports (3).

### 2.2 Good governance in local sports development: past, present and future

When we address the topic of managing sports organizations and good governance in sports, it is impossible not to mention leadership, ethics, transparency and accountability (13, 32, 33), as well as the modernization of management practices in terms of improving effectiveness and increasing efficiency (34), or the provision of sports services designed to meet the needs in the public sector (3).

However, despite the growing number of published studies and the considerable expansion of knowledge available to date (20, 35), we still face a scientific body that is not sufficiently broad and robust, given its importance (36). This difficulty is caused by the challenges of gathering relevant information and restrictions on access to properly published data, a reality common to the public and private sectors, both sporting and non-sporting (37, 38).

Regarding public sector, there is clearly greater interest in the activities and political intervention of local authorities, particularly due to the historical context of poor management and use of public resources (19), both in terms of transparency in using public funds and poor political and administrative management of the region under their jurisdiction, a reality that has been analyzed in several European countries throughout history (32). In this context, Portugal has also been subject to several studies that sought to analyze and produce knowledge regarding good governance in public organizations (39), especially regarding the activities of municipalities (39).

From a historical perspective, the development of sport in Portugal was marked by a major focus on sporting events during the period of dictatorship (1928–1933 and 1933–1974), with little concern for local public investment in promoting sport with the objective of encouraging physical exercise and sporting activities among the population (40). At that time, local authorities were subverted by the will and intentions of the central government, intervening very little in sport promotion (15, 40).

However, local sports development was also strongly influenced by the fall of the dictatorial regime, with the post-revolutionary period (41) being associated with a phase of major investment in the construction of specialized sports facilities throughout the country (41), along with the first local public sports services (8). After an initial phase, local authorities were given a wide range of legal powers and responsibilities delegated by the central government (13), giving local elected officials more autonomy and decision-making to develop the territory according to the population needs (13).

However, while there were a need and desire to develop the country quickly, this was not accompanied by adequate planning in terms of public investment and maintenance costs (3). This period was also marked by high local debt and inadequate sports facilities for the population in some regions (14). Most municipalities wanted to have their own swimming pool, athletics center, or sport arenas, in a kind of allusion to the “work done” by politicians, without properly assessing the real needs and future consequences for municipalities due to high indebtedness (3).

In terms of public governance and local sports development, the region was experiencing a positive period of development, but good governance was neglected. There was a lack of strategic planning, an absence of synergies and intermunicipal projects (14), to the point where many sports facilities canceled each other out due to their proximity (42). There were also some sports facilities that never opened due to construction irregularities, lack of demand, unsuitability for the characteristics of the population, or even lack of interest on the part of the resident population, demonstrating the absence of good governance for urban optimization and adequate management in the field of sports (41).

By joining the European Community, the scenario changed profoundly in terms of the responsibility of local authorities, as scrutiny became greater, along with the guidelines for political action. This required better management and decision-making based on prior studies or public consultations to bring citizens closer to decisions about territorial development, especially in urban centers (32, 34).

As a result of high levels of debt in the past, municipalities are now required to improve their capacity to manage and use public resources (43), while meeting the growing needs of their populations and responding to the demand for shorter modernization cycles (28). On the other hand, citizens are more aware, demanding, and involved in observing political action, being able to influence decisions and changes in political cycles based on their vision and perception of local development (16, 31).

The study of good governance in sports organizations is growing, including at the local level, although additional time and knowledge are required to consolidate it (3, 44). However, it seems clear that the future of good governance will involve the implementation of municipal management codes across the whole territory that respect efficiency, effectiveness, transparency, and ethics in the measures adopted (45–47), as well as evaluation instruments capable of collecting measurable data. This new approach will allow for the implementation of public sports policies to be correctly monitored and, if necessary, the initial objectives and assumptions to be corrected (3, 32, 34).

Good governance and transparency in sport require ongoing and comprehensive research, even though there has been enormous progress in this area of scientific knowledge. However, given the lack of investigations focusing on the implementation and monitoring of public policies, as well as local development by municipalities, it is essential to use alternative research methods to explore and generate new knowledge. One of the most used alternatives is to assess the opinions and perceptions of citizens, although little has been produced in the field of local sports development to measure the level of agreement and satisfaction with developments and progress in a particular region.

## 3 Methodology

Considering the lack of relevant data in the various sources of information available, this research is therefore exploratory in nature given the need to collect primary data to better understand a particular phenomenon. It follows a quantitative-descriptive approach for the treatment and presentation of results, using descriptive and inferential statistical methods to analyze the data collected.

### 3.1 Research design and participants

The purpose of the research was to assess the opinion/agreement of citizens residing in Portugal about local sports development according to different indicators. Therefore, to ensure the widest possible approach, while also aiming to achieve a representative sample of the universe under analysis, it was decided to conduct a national survey, including mainland Portugal, the Autonomous Region of the Azores and the Autonomous Region of Madeira.

To ensure the reliability and eligibility of responses, the study population was defined considering the number of voters registered on the electoral roll in December 2023, totaling 9,293,037 citizens. They are divided in 3 groups, i.e., Portuguese citizens, EU citizens (non-Portuguese) and other foreign citizens. This option ensured that responses were validated from citizens of legal age (+18) who were exclusively resident in Portuguese territory.

It was then decided that the study would be applied and conducted broadly, with the intention of disseminating it across a wide range of contexts within Portuguese society. Therefore, it was decided right from the start to include different types of sports organizations, both public and private, including public government institutes, sports federations and associations, foundations, sports clubs, private clubs and associations, associations representing different sports agents, trade unions, among others. On the other hand, non-sports public and private organizations were also included, including public and government organizations and institutes, foundations, associations, companies, higher education institutions, student associations, trade unions, among others.

The decision to conduct this multilevel survey across various sectors of society intended to ensure that the results would not be influenced by a greater affinity for sport, if only sports-related organizations had been considered. Diversity of participation was important, so the goal was to ensure that the results reflected the opinion/agreement not only of citizens with a greater connection to sport, but also those with less or no connection, in an attempt to achieve the highest possible level of neutrality and scientific rigor in the results obtained.

With the universe under study defined, data collection began using an online questionnaire which was sent by email to a total of 1,654 institutional contacts. To ensure that the sample was representative with a confidence level of 95% and a margin of error of 5%, it was necessary to obtain at least 385 responses. The questionnaire was available over a period of three months, with 971 responses received, although only 937 responses were validated after checking for inconsistencies.

Finally, we can safely assume that participants shared the invitations among their contacts, which makes it difficult to accurately assess the response rate. However, the high number of validated responses made it possible to achieve a representative sample of the population under study.

### 3.2 Procedures and data collection

Considering the exploratory nature of the research, as well as the lack of relevant data in the field under study, we immediately identified the need to collect primary data. We looked at whether there were any previously validated instruments that could be used or adapted, but we couldn't find any that fit the purposes of the study. Based on this, we decided to use a questionnaire specifically designed and structured to meet the study's objectives, having followed all the steps for its validation.

Recognizing that the main objective would be to reach as many citizens as possible, a simple and easy-to-understand structure was chosen, regardless of any differences in age, social context and education levels. On the other hand, an effort was made to use terms and expressions familiar to the context of sports, as well as those used by the media and other digital information sources.

Based on the established criteria, the questionnaire was structured into two sections, comprising a total of 7 questions and 12 statements. The first part aimed to collect sociodemographic data to characterize the profile of the respondents, while the second part of the questionnaire aimed to assess the level of agreement and opinion of citizens regarding sports development, transparency, and political priorities, being subdivided into two groups of statements. In total, the sociodemographic dimension consisted of 7 closed-ended questions, with the remaining 12 statements corresponding to the dimension “Municipalities, Sport and Society”. This last one was subdivided into two groups of 6 statements and intended to assess the level of agreement about different indicators based on a five-point Likert scale, where 1 meant “I completely disagree” and 5 meant “I completely agree”.

A pre-test was carried out involving 12 citizens from different professional backgrounds, as well as 3 experts from academia, which allowed us to gather suggestions for optimization and make minor adjustments in statements. Next, the internal consistency of the scales was verified using Cronbach's alpha statistical measure to assess the reliability, correlation, and homogeneity of the items, with the reliability coefficient being reasonable (between *α* > 0.708 and *α* > 0.722). The final version was also validated by the Ethics Committee of the University of Évora (Portugal).

As mentioned above, the questionnaire aimed primarily to measure citizens' level of agreement and proximity with various statements closely related to local sports development, such as satisfaction/perception of the level of sports development, provision of sports services and facilities, priorities, transparency, sports programs, among others, to assess their opinion/perception about the current state of the municipality where they live.

The data was collected between November 2024 and January 2025, obtaining 937 validated responses. The objectives of the study were presented to all recipients, as well as the authors' name and affiliation. The confidentiality of the responses was also ensured, since no personal data were collected during the survey process. The data collection complied with all ethical procedures set out in the European Code of Conduct for Research Integrity, as well as the good research practices regulated by the University of Évora (Order No. 89/2023). Before finishing the questionary, voluntary participation was requested to validate the responses. To ensure that the questionnaire was not accessed more than once, we activated the option to allow only one response per user.

The study was communicated solely by email, with an institutional disclosure presentation accompanying the link to the questionnaire and recipients were selected at random, with no exclusion criteria in order to ensure homogeneity between sports and non-sports organizations from a wide range of social contexts. Considering that requests for participation were sent to the institutional contacts available online, we conceive the possibility that they have been disseminated internally. Of the 1,654 requests sent, 31% (*n* = 513) were unsuccessful, mainly due to “address not found.”

### 3.3 Data analysis

To ensure the best use of the data collected and considering the exploratory nature of the research, descriptive and inferential statistical methods and techniques were used in the data analysis, using SPSS 30.0 software to process and present the results in tables.

The data was analyzed according to the two dimensions of the questionnaire, with the following variables defined in the sociodemographic dimension: a) gender; b) age; c) academic qualifications; d) nationality; e) professional status; f) region of residence; and g) sport practice habits. In the dimension called “Municipalities, Sport and Society”, the following variables were defined: a) level of sports development; b) provision of sports programs and services; c) sport venues and facilities; d) communication; e) transparency; and f) political priorities.

In the descriptive analysis, we used parameters for the distribution of variables, namely, frequency, percentage, mean, and standard deviation. To perform the inferential analysis, considering that the variables did not show a normal distribution when applying the Kolmogorov–Smirnov test, nonparametric tests were applied. To compare the variables under study between two groups, the Mann–Whitney test was applied, as it is the most appropriate for comparing distribution functions of at least one ordinal variable measured in two independent samples. On the other hand, to compare three or more independent groups in relation to an ordinal or continuous quantitative variable, the Kruskal–Wallis test was applied, followed by Bonferroni's multiple comparison test to compare pairs of groups for greater statistical accuracy.

## 4 Results

The results are divided into two sections, beginning with a characterization of the respondents, followed by the results on Municipal Sports Development, Transparency and Political Priorities.

### 4.1 Sociodemographic profile of respondents

Opinion polls generally tend to relativize the profile of respondents, since their main objective is to capture opinions/perceptions about a particular phenomenon. It is therefore important to characterize the citizens involved in this study about the local development of sport. According to Table 1, it is immediately visible that participation was homogeneous in terms of gender, with a slight predominance of males (*n* = 486, 51.9%).

**Table 1:** Sociodemographic profile.

Sociodemographic variables	Male		Female	
	*f*	%	*f*	%
Gender	486	51.9%	451	48.1%
Age range (by gender)				
18–25	28	5.8%	37	8.2%
26–35	57	11.7%	76	16.8%
36–45	135	27.8%	105	23.3%
46–55	152	31.3%	160	35.5%
56–65	93	19.1%	68	15.1%
>65	21	4.3%	5	1.1%
Total	(486)	(100.0%)	(451)	(100.0%)
Academic qualifications				
Basic Education	12	2.5%	6	1.3%
Secondary Education	115	23.7%	95	21.6%
Bacheloŕs Degree	192	39.5%	186	41.2%
Masteŕs Degree	123	25.3%	91	20.2%
Ph.D. Degree	44	9.0%	73	16.2%
Total	(486)	(100.0%)	(451)	(100.0%)
Region of residence				
Mainland Portugal				
North	100	20.6%	66	14.6%
Centre	122	25.1%	96	21.3%
Lisbon Metropolitan Area	199	40.9%	226	50.1%
Alentejo	28	5.8%	24	5.3%
Algarve	13	2.7%	22	4.9%
Autonomous Region of the Azores	12	2.5%	14	3.1%
Autonomous Region of Madeira	12	2.5%	3	0.7%
Total	(486)	(100.0%)	(451)	(100.0%)
Sports practice habits				
Practice	339	69.7%	284	63.0%
Non-practice	147	30.3%	167	37.0%
Total	(486)	(100.0%)	(451)	(100.0%)

In terms of age, it was observed that most male respondents were between 36 and 45 years old (*n* = 135, 27.8%) and between 46 and 55 years old (*n* = 152, 31.3%). In the case of female respondents, it was observed that the two previous groups are also the most representative, namely those aged 36–45 (*n* = 105, 23.3%) and those aged 46–55 (*n* = 160, 35.5%).

Regarding marital status, it was observed that among male and female respondents, the vast majority were married (*n* = 441, 47.1%), followed closely by the single group (*n* = 255, 27.2%). In terms of professional status, the vast majority were employed (*n* = 786, 83.9%), followed by self-employed workers (*n* = 69, 7.4%) and students (*n* = 37, 4.0%). Of the 937 responses, 99.1% (*n* = 929) of respondents were Portuguese nationals, 0.6% (*n* = 6) were European nationals, and 0.4% (*n* = 3) were other foreign nationals.

In terms of academic qualifications, it was observed that most male respondents had higher education qualifications (*n* = 359, 73.8%), a pattern also observed among female respondents (*n* = 350, 77.6%). The level of education was mainly at the bachelor's degree level (M = 192, 39.5% vs. F = 186, 41.2%), with 16.2% (*n* = 73) of female respondents having a doctorate, while among males this percentage was only 9.0% (*n* = 44).

As for the region of residence, it was observed that most respondents lived in the Lisbon Metropolitan Area (*n* = 425, 45.4%), followed by the Central region (*n* = 218, 23.3%) and the North region (*n* = 166, 17.7%). Despite the relatively higher number of responses in the Lisbon Metropolitan Area, possibly due to the greater concentration of citizens willing to participate in this type of survey, the distribution of responses is consistent with Portugal's demographic structure. Approximately 28.0% of the resident population in Portugal lives in the Lisbon Metropolitan Area, while 21.7% and 34.6% live in the Center and North regions, respectively, even though the territorial size of the last two is much larger when compared to the Lisbon Metropolitan Area.

Finally, it was considered important to assess the status of respondents as sports practitioners or non-practitioners. The results demonstrate that most citizens declared that they were sports practitioners, with higher percentage among males (*n* = 339, 69.7%) compared to females (*n* = 284, 63.0%). Even so, 33.5% indicated that they were not sports practitioners.

### 4.2 Municipalities, sport and society

The main objective of the study was to assess the level of agreement and satisfaction of citizens with local sports development. Therefore, the results of this dimension are organized into four tables, in order to present the analyses carried out across the various indicators covered.

As shown in Table 2, it is immediately evident that most respondents (*n* = 536) agreed (*n* = 329, 35.1%) and completely agreed that the municipality where they live values local sports development, something we consider very relevant. As for the public offer of sports services, spaces, and facilities, the majority agreed (*n* = 331, 35.4%) and completely agreed (*n* = 138, 14.7%) that the existing offer is adequate. Even so, there was a considerable number of responses among those who neither agreed nor disagreed (*n* = 265, 28.3%), which may be due to a lack of knowledge on this issue.

**Table 2:** Levels of agreement on municipal sports development.

I completely disagree		I disagree		I neither agree nor disagree		I agree		I completely agree	
*f*	%	*f*	%	*f*	%	*f*	%	*f*	%
The municipality where you live values local sports development.									
26	(2.8%)	102	(10.9%)	273	(29.1%)	329	(35.1%)	207	(22.1%)
The municipality where you live has an adequate public offer of sports services, spaces and facilities.									
48	(5.1%)	155	(16.5%)	265	(28.3%)	331	(35.4%)	138	(14.7%)
The municipality where you live regularly develops local sports initiatives and activities that are open to the public.									
28	(3.0%)	142	(15.2%)	277	(29.6%)	322	(34.4%)	168	(17.8%)
The municipality where you live regularly publishes information about available sports activities and programs, as well as one-off events that take place outside the municipal sports annual program.									
41	(4.4%)	169	(18.0%)	282	(30.2%)	290	(30.9%)	155	(16.5%)
You consider the level of political action and local development of sport when you vote in local elections.									
75	(8.0%)	138	(14.7%)	289	(30.8%)	266	(28.4%)	169	(18.1%)
Globally, you are satisfied with the level of sports development in the municipality where you live.									
51	(5.4%)	170	(18.2%)	298	(31.8%)	320	(34.1%)	98	(10.5%)

Regarding regular development of public sports initiatives and activities promoted by the municipality, the vast majority (*n* = 490) of respondents agreed (*n* = 322, 34.4%) and completely agreed (*n* = 168, 17.8%) with the statement, recognizing this action on the part of the municipalities, even though a considerable number of people still neither agree nor disagree (*n* = 265, 28.3%).

In terms of regular communication of sports activities and programs developed by municipalities, there is a dispersion of responses, since 47.4% (*n* = 445) agreed with the statement, 30.2% (*n* = 282) neither agreed nor disagreed, and 22.4% (*n* = 210) disagreed, making it impossible to accurately assess the level of satisfaction, even though the number of responses is higher in the “I agree” and “I completely agree” fields.

From a different perspective, we intended to assess whether the opinion about local sports development could (or could not) influence citizens' votes in local elections, with most respondents neither agreeing nor disagreeing (*n* = 289, 30.8%). However, when considering the remaining options, we found that 46.5% (*n* = 435) admitted considering this issue when voting, compared to 22.7% (*n* = 213) who did not agree with this statement. If we consider that sport has never been studied as a deciding factor in the choice of elected politicians, we can say that these results require a more careful analysis, while arousing curiosity.

Finally, when asked whether they were generally satisfied with the level of local sports development in their municipality of residence, most respondents agreed (*n* = 320, 34.1%) and completely agreed (*n* = 98, 10.5%) with the statement, while 23.6% (*n* = 221) of citizens disagreed. Although there is some dissatisfaction with local sports development in their region, most citizens are satisfied.

Regarding the level of agreement on transparency and political priorities in municipal sports, we can observe in Table 3 that in terms of transparency, most citizens neither agree nor disagree (*n* = 397, 42.4%) with the statement that there is transparency in municipal political action regarding investment in sport and how it is implemented. The responses among those who agree with the statement (*n* = 289, 30.8%) and those who disagree (*n* = 251, 26.8%) are very similar, which makes it difficult to clarify the results based on citizens' opinions.

**Table 3:** Levels of agreement on transparency and political priorities.

I completely disagree		I disagree		I neither agree nor disagree		I agree		I completely agree	
*f*	%	*f*	%	*f*	%	*f*	%	*f*	%
The municipality where you live is transparent about its investment in sport, publicly disclosing the reasons for and manner of municipal public spending.									
93	(9.9%)	158	(16.9%)	397	(42.4%)	196	(20.9%)	93	(9.9%)
Improving conditions and access to sports should be one of the priorities of local political action.									
4	(0.4%)	26	(2.8%)	92	(9.8%)	296	(31.6%)	519	(55.4%)
The promotion of physical activity and the development of sport should be a political priority based on the creation and adoption of public policy measures and instruments that benefit the population in its various age groups and contexts, as well as through public investment and support for various sports agents.									
4	(0.4%)	19	(4.1%)	170	(18.1%)	349	(37.2%)	395	(42.6%)
Public investment in sports should prioritize local sports development through school sports, sports associations, and adequate public spaces, with the main objective of increasing the number of active participants, as well as creating and adopting healthy habits and improving the physical condition of the population.									
7	(0.7%)	16	(1.7%)	126	(13.4%)	276	(29.5%)	512	(54.6%)
The municipal public investment in sports promotes and boosts local economic, cultural, social, and tourist development.									
24	(2.7%)	56	(6.0%)	180	(19.2%)	328	(35.0%)	349	(37.1%)
There are differences in municipal public investment in sport depending on political party orientation.									
41	(4.3%)	68	(7.3%)	413	(44.1%)	267	(28.5%)	148	(15.8%)

As for political priorities, we observe that the vast majority completely agreed (*n* = 519, 55.4%) that improving conditions and access to sports should be one of the local political priorities. If we also consider the citizens who agreed (*n* = 296, 31.6%), we see that 87.0% (*n* = 815) of respondents agreed with the statement, leaving no room for doubt, since only 3.2% (*n* = 30) disagreed. Regarding the promotion and development of physical activity and sports by municipalities, the vast majority agreed (*n* = 349, 37.2%) and completely agreed (*n* = 395, 42.6%) that the adoption of public policies should benefit the entire population without exception, as well as supporting public investment in the various sports agents in the region. It should be noted that only 4.5% (*n* = 23) disagreed.

In terms of municipal public investment in sport, the vast majority (*n* = 788, 84.1%) unanimously agreed that this investment should prioritize sports development through school sports, sports associations and clubs, as well as the creation of adequate spaces for sports practice, in order to contribute to the adoption of healthier habits by citizens. Only 2.1% (*n* = 23) disagreed with this statement. Regarding the value of municipal public investment in sport, the majority agreed (*n* = 328, 35.0%) and completely agreed (*n* = 349, 37.1%) that this investment promotes local development in economic, cultural, social, and tourism terms, in contrast to the 8.7% (*n* = 80) who disagreed.

Finally, we intended to assess whether there were differences in the way municipal public investment in sport is made depending on political orientations, with most citizens (*n* = 413, 44.1%) neither agreeing nor disagreeing with the statement. However, the group that agreed was significantly larger (*n* = 415, 44.3%) than those who disagreed (*n* = 109, 11.6%). This may help to clarify the potential of these results in the citizens' assessment of different political party orientations.

Regarding transparency, we tried to explore the possible relationship between investment in sport and the prioritization of local sports development with the variables of age and academic qualifications.

In this sense, Table 4 shows that, of the correlation between age, education, transparency regarding investment in sport, and prioritization of local sports development, the results indicate that prioritization of local sports development is positively associated with age (*r* = 0.135) and education (*r* = 0.198), both correlations being significant at the 0.01 level. This means that older participants with higher levels of education tend to attach greater importance to prioritizing local sports development, while the variable relating to transparency of municipal investment did not show any associations.

**Table 4:** Correlation between age, education, transparency, public investment and local priorities.

Transparency, public investment and local priorities in sport	Age	Academic qualifications
The municipality where you live is transparent about its investment in sport, publicly disclosing the reasons for and manner of municipal public spending.	−0.011	0.016
Public investment in sports should prioritize local sports development through school sports, sports associations, and adequate public spaces, with the main objective of increasing the number of active participants, as well as creating and adopting healthy habits and improving the physical condition of the population.	**0.135** **	**0.198** **

** The correlation is significant at the 0.01 level (two-tailed).

Finally, we considered it to be relevant to analyze and compare the relationship between sports practice, age, recognition of local sports development, adequate public provision of services, sports facilities and venues, and regular dissemination of information about sports activities and programs.

Therefore, it is possible to observe in Table 5 that age revealed very similar averages between both groups, suggesting that sports practice is not differentially associated with the age of the respondents.

**Table 5:** Correlation between age and perceptions of sports development and services based on sports participation.

Local sports development and sports participation	Engaging in physical or sports activities						*P*
	No			Yes			
	N	Mean	SD	N	Mean	SD	
Age	314	45.8	11.6	623	45.0	12.1	0.401
The municipality where you live values local sports development.	314	3.6	1.0	623	3.7	1.0	0.326
The municipality where you live has an adequate public offer of sports services, spaces and facilities.	314	3.4	1.1	623	3.4	1.1	0.688
The municipality where you live regularly publishes information about available sports activities and programs, as well as one-off events that take place outside the municipal sports annual program.	314	3.3	1.1	623	3.4	1.1	0.191

On the other hand, perceptions about whether the municipality values local sports development, provides adequate public facilities, and disseminates regular information about sports activities and programs were similar among practitioners and non-practitioners. Overall, the practice of physical or sporting activity does not seem to influence participants' perceptions of the municipality's role in the development of sport, nor is it associated with significant differences in age.

## 5 Discussion

The study presented is part of existing research on sports development and its characterization from a multidisciplinary perspective and analysis in the field of sport management (48).

Considering the lack of data about the historical evolution of local sports development, as well as results on society perception in this area, the main objective was to assess the level of agreement and satisfaction among Portuguese citizens about sports development in the municipality of residence. To achieve this, several indicators representing the most characteristic elements when assessing sports development were tested and analyzed (3, 14).

Local development and municipalities have been extensively studied by different authors and perspectives (4, 8, 49). Nevertheless, there is a consensus that this is an area of knowledge that clearly needs to be explored in greater depth, given the relevance, multiple functions and fields of activity of municipalities, as well as their socioeconomic impact on the citizens daily lives (49, 50).

The difficulties most frequently cited by researchers are the lack of instruments for evaluating public policies and the absence of relevant data; the lack of transparency and resistance to the disclosure of activity reports and political evaluations of measures adopted; and the lack of available information to public access, often concealed by the municipalities (51, 52), as well as the possible lack of interest by citizens in opinion polls, which relieves local politicians of their accountability (37, 53).

When it comes to local sports development, we see that the most scientific approaches fall into: a) characterization of public policies and municipal activity, including sports services and facilities, as well as the associative movement; b) leadership and professionalization of sports agents, including in the public sector; c) municipal public investment in sports activities; and d) sporting events as a factor in boosting and promoting physical and sporting activities, as well as the local economy and tourism, among others (3, 54, 55). On the other hand, no relevant studies were identified in which citizens were surveyed about their opinion/satisfaction with the local development of sport.

Difficulties are also pointed out in obtaining relevant information on municipal activity and in public access to technical-scientific reports, which would allow for more detailed analyses (3, 56). In practice, there is growing political interest in sport, especially in national and international sporting success, which contrasts with the willingness and intention to evaluate local sports development measures and publicly disseminate their results for a better understanding of the phenomenon (57).

Overall, and despite the lack of data enabling a close comparison, the profile of respondents follows a similar pattern in opinion surveys, particularly about gender, age group, and level of academic qualifications (58, 59). The results have showed a trend in existing different contexts where citizens who are more academically educated, live in urban centers, and are over 35 years of age are more likely to participate in opinion/perception studies related to society topics (60).

Two points that we consider key are the existing barriers and a certain resistance in citizen participation on issues that directly impact them (61), essentially due to a lack of interest or because they do not foresee any usefulness in their opinion in improving a particular phenomenon (62). This evidence, previously highlighted by other authors, corroborates with the results obtained regarding the high number of responses “I neither agree nor disagree”. Public opinion really matters when it comes to how people see politics and political leaders (31), influencing what they do (30), so you'd expect people to be more interested.

The second key point particularly relevant to a better understanding of this study is related to the number of citizens who reported practicing sports (*n* = 623, 66.5%). When compared with previous data (3), there has been an increase in the number of citizens who identify themselves as sports practitioners, which may be related to the level of local sports development and improvements in accessing physical and sporting activities in cities, as suggested by some authors (3, 62, 63). Although the study cannot establish a cause-effect relationship, the results show that municipalities continue to be the main agents in promoting and fostering the development of sport (8, 13).

Considering that we sought to analyze opinions/perceptions among the population about local sports development, we find particularly curious the possible relationship between the number of citizens who identified themselves as sports practitioners, a trend that is apparently growing when compared to previous studies (3, 12, 64, 65), and the percentage of responses “I neither agree nor disagree”, as we consider this type of response to demonstrate an uninformed opinion on a given subject.

By analyzing the results in Table 2, for example, the percentage of responses of “I neither agree nor disagree” varies between 28.3% and 31.8%, while in Table 3 it varies between 9.8% and 44.1%, which from the point of view of science, raises questions on why the responses “I neither agree nor disagree” were given. Was it due to a lack of knowledge? Disinterest? Not having enough information? Other reasons? Although the results do not allow us to adequately evaluate this hypothesis, we consider this occurrence to be a phenomenon in itself and an opportunity for further study, mostly because it is an investigation that sought to assess perceptions and opinions about sports development.

While there is some data showing an overall decrease in the number of federated athletes in Portugal (64), a situation that has little to do with the intervention of municipalities (indirect action), it is also recognized that the increase in non-competitive sports for leisure, recreation, or improving physical condition and health is strongly associated with improved conditions for access to and practice of sports in cities (3, 66), with municipalities playing the main role in this increase due to their direct intervention (6) and proximity to the real needs of the population and the territory.

In this sense, it would be expected that citizens' greater proximity to local political action and the development (or lack thereof) of cities would promote a greater awareness of the evolution of sports development, especially in terms of the creation and improvement of public spaces and areas for leisure and sports, but also in relation to a greater or lesser awareness of local sports activities, the promotion of sporting events, etc., regardless of whether the opinion is positive or negative according to each context. While not being a practitioner and/or close to sport could justify the absence of a formed opinion, we tend to consider that in the case of sports practitioners, greater awareness in this regard would be expected and that this would translate into a formed opinion.

While it is true that there is not always a correlation between literacy, political awareness, and development (67, 68), it is a fact that, in general, people today are more informed about topics that interest them and seek concrete answers in the field of sports that are consistent with their motivations and priorities (69, 70). If we consider the development of sport and the role of local authorities in promoting and investing in sport, it would be reasonable to expect that greater proximity could lead to more informed opinions, whether positive or negative, but the results do not clearly support this hypothesis.

Regarding local sports development, it was observed that most citizens (57.2%) considered that their municipality values sports, with 50.1% of citizens stating that there is adequate public provision of sports services, spaces, and facilities. On the other hand, 52.2% of citizens agreed that their municipalities regularly develop sports initiatives and activities that are open to the public, with 47.4% agreeing that municipalities regularly disseminate diverse information about sports programs, events, or activities.

However, when asked about the overall opinion regarding the level of local sports development, only 44.6% agreed that they were satisfied, with a high proportion of citizens having no opinion (31.8%). We consider the number of responses to each statement by citizens without a formed opinion to be relevant, reinforcing the findings of other authors that disinterest in or lack of knowledge about a particular reality is the main reason for non-participation and/or absence of a formed opinion (71).

Given the relevance of other social issues and concerns, it was observed that 46.5% of citizens consider the level of political action and local sports development in local elections. We consider this output as an extremely relevant fact that has not yet been surveyed in the study on this subject, even considering that 31.8% do not express a formed opinion. This factor should be interpreted by municipalities as a highly relevant indicator in terms of political assessment and its impact on citizens' voting intentions, whether positive or negative, which may determine whether local elected officials remain in power or not.

Regarding the political priorities of municipalities in the field of sport, there was broad consensus on where and how municipal action strategies should be focused, with these priorities being in line with the opinion of citizens and European guidelines (3, 4, 36), as well as with the European Model of Sport (3). The promotion of sport in Europe is mainly ensured by public intervention from Member States at various levels through the adoption and definition of public policies (6) for the promotion, support, and improvement of regular physical activity and sport. Through individual and collective experiences of regular sports practice, sport stands out as a multidisciplinary tool for acquiring social skills that are reflected in more organized societies with humanistic and progressive values (72), reinforcing sport as a pillar of human development (24, 73).

Citizens have recognized the importance of public policies that prioritize safe, secure, and healthy public access to sports for all without exception (73) through support for school sports, recreational and sports associations, and investment in public sports services, spaces, and facilities that are accessible and offer equal opportunities, as well as regular municipal initiatives and programs. These priorities had already been identified by other authors (74, 75), meaning that the results obtained reinforce this vision, as Portuguese citizens (55.4%) clearly stated that this should be one of the priorities in local sports policies.

Municipal investment in sport is also recognized by 72.1% of citizens for its impact in generating value in the development of the local economy, culture, social integration and cohesion, and tourism, a factor that has already been also validated by several authors (3, 49, 58). According to 44.3% of citizens, this investment is carried out differently depending on the political orientation of local elected officials, which is an opportunity for further study.

Transparency in municipal activities has been deeply studied in recent years, particularly about accountability, good public management of resources, and the implementation of measures to combat corruption, opacity, and the disclosure of publicly accessible information (27–29). These issues have been developed by national and international studies, allowing for a more rigorous assessment and analysis of transparency indices, even though most results point to areas for improvement in relation to leadership, transparency and accountability (75, 76), as well as for more concise communication and disclosure regarding political activity.

When it comes to public opinion, different authors say that there is a high level of detachment from these issues (77, 78), which makes it hard to push for more efficient, effective, and replicable control tools that can be applied to a wide range of municipal situations (79, 80). The results obtained, although not very in-depth in terms of indicators of transparency in municipal action, point in the same direction, since most citizens (42.4%) indicated that they had no opinion on the existence of transparency in their municipality in relation to public investment in sport, with the percentage being very similar between those who agreed (30.8%) and those who disagreed (26.8%).

While political priorities are influenced by age and level of academic qualifications, indicating that the older and more educated people are, the greater the importance they attach to priorities in local sports development. Transparency, on the other hand, does not show a significant association. In other words, even citizens with higher levels of academic qualifications, who tend to be more alert and knowledgeable about the importance of greater transparency in municipal actions, show a certain detachment and reinforce the idea that society still shows little interest in subjects they do not control (81), even if they have direct implications in their daily lives and good governance of their municipality.

Finally, statistically significant differences between practitioners and non-practitioners in relation to their perception of the level of local sports development would be expected, but the results point to the opposite scenario, i.e., citizens who practice sports do not express a different or stronger opinion about the performance of municipalities. This reality contrasts with results obtained in other contexts, where the difference between practitioners and non-practitioners suggested a deeper knowledge of different aspects related to the development and importance of sport (82–84). If we add to this question the high number of responses obtained without a formed opinion, we find that there is a whole field of study that needs to be explored in greater depth to better understand the relationship between those who practice sports and the social involvement that sports provide.

## 6 Conclusions

The relationship between the importance of municipalities in empowering and promoting sport and the existing scientific knowledge is still very deficient, especially in Portugal. The study offers a pioneering approach by providing insight into the perceptions of citizens and their satisfaction with municipal action in their area of residence, which has not yet been developed in this context. In modern societies, the extent of this opinion may even influence political decisions, putting pressure on politicians to respond and listen to citizens more closely and effectively.

The results suggest that municipalities are fulfilling their role in local sports development as recommended in their legal duties and powers, with citizens recognizing the progress made throughout the years since the establishment of the democratic regime. Their opinion also contributes as an acknowledgement of the work carried out, legitimizing political action and indicating that the progress made has been widely accepted, and that continuity and investment in meeting the needs of territories and their populations in terms of access to physical activity and sport in conditions of safety, protection, and equality for all citizens without exception should therefore be pursued.

In addition, it is highly advisable to encourage municipalities to adopt measures of good governance and transparency when defining structural public policies, since they continue to play a fundamental role in regional development, with a particular emphasis on the development of sport and the empowerment of society. Finally, results also reveal a high percentage of population without a formed opinion, which may indicate indifference or an inability to decide due to a lack of agreement, whether positive or negative. Elected politicians should seek information on whether the measures are reaching the majority of the population or only a part of it.

## 7 Limitations and future research

The lack of scientific and socio-political surveys about the evolution of local sports development did not allow for the desired overall comparison of society's opinion/perception on this issue. Furthermore, the lack of responses from regions whose participation was lower than expected did not allow for a comprehensive and more homogeneous evaluation. A wider distribution across the sample would have been important to better understand the opinion/perception regarding sports development in municipalities with more specific characteristics (smaller cities, rural areas, etc.).

Ultimately, the results provided greater detail on the previously unknown opinion/perception of citizens in regions with larger resident populations but were limited to more structural conclusions regarding the overall spectrum of the various regions of Portugal. Overall, these discrepancies did not affect the initial purpose of the study, as they produced new knowledge. However, it is recommended that a new approach focuses primarily on regions that have registered a lower participation rate.

Future research aimed at contributing to this field, as well as serving as a tool for the implementation of appropriate public policies focused on citizens' needs by policy makers, should reinforce the intention to regularly gather the opinion of local population. This allows for future comparisons and historically charts the evolution of satisfaction with local sports development. A refined assessment of opinion on specific areas of local sports development is also suggested to introduce new indicators for the collection of more detailed data, for example, exploring satisfaction with the municipal sports services available in each region and their characteristics.

## Data Availability

The raw data supporting the conclusions of this article will be made available by the authors, without undue reservation.
